# Is Genetic Testing of HER2-Negative Metastatic Breast Cancer Patients Implemented into Clinical Practice? A Retrospective Analysis

**DOI:** 10.3390/jcm15093433

**Published:** 2026-04-30

**Authors:** Christine Deutschmann, Florian Heinzl, Carmen Leser, Daphne Gschwantler-Kaulich, Christian F. Singer, Suncica Kostic, Adelheid Golescu, Georg Pfeiler

**Affiliations:** Department of Obstetrics and Gynecology, Division of General Gynecology and Gynecologic Oncology, Medical University of Vienna, Waehringer Guertel 18-20, 1090 Vienna, Austria

**Keywords:** genetic testing, HER2− metastatic breast cancer, PARP inhibitor, platinum salt

## Abstract

**Background/Objectives**: Genetic testing in Human Epidermal Growth Factor Receptor 2-negative (HER2−) metastatic breast cancer (mBC) is necessary to enable optimal treatment choices including poly(ADP-ribose)polymerase inhibitors (PARPis). The present study evaluated the implementation of genetic testing in a real-world setting to reveal and subsequently allow targeting of potential inadequacies and risk factors for low testing frequency. **Methods**: We performed a retrospective analysis including HER2− mBC patients treated at a single academic center starting from 10 April 2019 (date of European Medicines Agency (EMA) approval of Olaparib for germline breast cancer gene mutant (gBRCAm) HER2− mBC) to 7 September 2021. The primary objective of the study was to evaluate the rate of HER2− mBC patients that were recommended to undergo genetic testing by the multidisciplinary tumor board (MTB). The secondary objective was to identify factors that were associated with a higher likelihood of having undergone genetic testing. **Results**: In total, 47.6% (109 of 229) of HER2− mBC patients had been recommended to undergo genetic testing by the MTB. Of these informed patients, 89.0% (97 of 109) underwent genetic testing, of which 11.6% (11 of 95) had a germline BRCA mutation (gBRCAmut) and were eligible for PARPi treatment. In multivariate analysis, younger age (*p*-value: 0.0007), hormone receptor positive (HR+)/HER2− subtype (*p*-value < 0.0001) and positive family history for breast and ovarian cancer (*p*-value: 0.0001) were significantly associated with the performance of genetic counseling. **Conclusions**: The present study demonstrated low genetic counseling rates of HER2− mBC patients, especially in individuals without specific risk factors for hereditary breast cancer. Informed patients showed a high willingness to undergo genetic testing. Genetic testing revealed targetable mutations in over 10% of tested patients.

## 1. Introduction

In breast cancer gene (BRCA)-deficient breast tumors with malfunctioning homologous recombination, poly(ADP-ribose)polymerase inhibitors (PARPis) prevent the repair of single-strand deoxyribonucleic acid (DNA) breaks resulting in the accumulation of double-strand breaks and stalled DNA replication forks subsequently leading to tumor cell death [1].

The two pivotal trials OlympiAD and EMBRACA demonstrated a significantly longer median progression free survival (PFS) with the PARPis Olaparib and Talazoparib versus treatment of physicians choice (TPC) with consistent results across various subgroups [2,3].

No benefit in overall survival (OS) with Olaparib or Talazoparib versus TPC was observed. Yet, significant cross-over following progression from placebo to PARPi might have confounded OS results [4,5].

In both OlympiAD and EMBRACA significant improvement in patient-reported quality of life with a greater delay in time to clinically meaningful deterioration was reported by patients receiving a PARPi versus TPC [2,6].

Up to 10% of patients with metastatic Human Epidermal Growth Factor Receptor 2-negative (HER2−) breast cancer (mBC) have a BRCA mutation (BRCAmut) with an even higher prevalence (>30%) in triple negative breast cancer (TNBC) and are therefore eligible for PARPi treatment [7,8,9,10]. Notably, more patients with a BRCAmut have hormone receptor positive (HR+), HER2− disease as this subtype is more prevalent compared to TNBC.

The advent of PARPis has led to their inclusion in all established international treatment guidelines and the recommendation for genetic testing in HER2− mBC patients [11,12,13].

Furthermore, international guidelines include platinum agents (cisplatin and carboplatin) as preferred treatment options in recurrent unresectable or mTNBC with a BRCAmut [12].

Preclinical studies demonstrated a high sensitivity of cells lacking functional BRCA1 or BRCA2 to cisplatin owing to the formation of covalent crosslinks in the DNA and an impaired ability to repair this damage in the absence of homologous recombination [14,15].

The TNT phase III study showed significant improvements in the objective response rate (ORR) (68% versus 33%, *p* = 0.03) and PFS (6.8 versus 4.8 months, *p* = 0.03) with carboplatin versus docetaxel in germline BRCA mutant (gBRCAm) recurrent, locally advanced or mBC [16]. The interaction between the treatment effect regarding the ORR (*p* = 0.01) as well as PFS (*p* = 0.03) and BRCA status was significant [17]. Notably, it is not clear how platinum agents compare with PARPis and should be sequenced in this setting [12].

Given the advent of platinum salts as well as PARPis in BRCAm HER2− mBC timely genetic testing in these patients is of high importance to allow adequate treatment [2,3,18,19,20,21,22]. Optimal therapeutic decisions are of particular importance in HR+/HER2− BC with a BRCAmut as this patient population has shown decreased OS compared to BRCA wild-type (WT) (BRCA1m: *p* = 0.0008; BRCA2m: non-significant) [23].

Yet, testing rates in these patients have been reported suboptimal varying between 16.7% to 97% [24,25,26,27] and being lowest in older patients, those with HR+ mBC and those without a known family history of breast or ovarian cancer [28]. Furthermore, in a real-world international study including the USA, France, Germany, Italy, Spain and the UK BRCA 1/2mut testing rates were significantly less frequent in the European countries than in the USA [28].

The present study evaluated the implementation of genetic testing in a real-world academic setting as well as factors associated with a higher likelihood of having undergone genetic testing. With this study potential deficiencies in genetic testing are revealed which will in the future allow the introduction of measures to increase genetic testing and specifically target populations at risk for low testing rates.

## 2. Patients and Methods

The primary objective of the present study was to evaluate the rate of HER2− mBC patients treated at a single academic center in Austria that were recommended to undergo genetic testing by the multidisciplinary tumor board (MTB). The secondary objective of the study was to evaluate factors that were potentially associated with a higher likelihood of having undergone genetic testing including age, BC subtype, treatment line for the metastatic setting and family history.

Age and treatment line were assessed at the time of genetic counseling or if genetic counseling was not performed at the time of last follow-up. BC subtype was assessed according to the latest tumor biopsy. Positive family history was defined as the occurrence of multiple cancer cases in one family line or special situations including at least three BC cases; at least two BC cases with one case occurring prior to 51 years of age; at least two ovarian cancer cases; at least one BC and one ovarian cancer case; at least one woman with breast and ovarian cancer; at least one case with bilateral BC with the first diagnosis prior to 51 years of age; at least one male BC case; a known mutation in BRCA1, BRCA2, ATM, BARD1, BRIP1, CHECK2, PALB2, RAD51C or RAD51D in the family; a personal history of early-stage HER2− BC with high risk of recurrence; or high-grade epithelial ovarian cancer.

We included all HER2− mBC patients treated at the Department of Obstetrics and Gynecology of the Medical University of Vienna, Austria, starting from 10 April 2019 (date of European Medicines Agency (EMA) approval of Olaparib for gBRCAm HER2− mBC) to 7 September 2021. Metastatic disease was assessed centrally by computer tomography (CT) and biopsy of a metastasis in the course of routine clinical care. There were no exclusion criteria.

We conducted a retrospective chart review and assessed whether genetic counseling was recommended by the MTB and testing was subsequently performed, as well as patient and disease characteristics.

At the study site genetic counseling and blood collection for germline genetic testing were conducted by a gynecologist trained in genetic counseling. In the course of genetic testing 18 genes including ATM, BARD1, BRCA1, BRCA2, BRIP1, CDH1, CHEK2, EPCAM, MLH1, MSH2, MSH6, PALB2, PMS2, PTEN, RAD51C, RAD51D, STK11 and TP53 were evaluated using next-generation sequencing (NGS). In one patient of the study genetic testing was solely performed on tumor tissue (somatic testing).

In the course of a subsequent study all patients who had not been advised to undergo genetic testing by the MTB as well as patients who had been recommended to undergo genetic testing by the MTB but had not for an unknown reason were contacted and invited to undergo genetic testing (end of patient recruitment: 8 May 2024). The testing rates of these patients are presented in the present manuscript to allow thorough discussion of patients’ acceptance to undergo genetic testing. Information on genetic test results of these patients and uptake of PARPi treatment is still pending at the time of manuscript preparation and will be presented elsewhere.

Statistical analysis was done using R version 4.3.2 and package viridis version 0.6.4. Categorical data are presented by absolute and relative frequencies, whereas numerical data are aggregated by mean and standard deviation (SD). Differences between groups were tested with a chi-squared test for the former and Student’s t-test for the latter. ‘Genetic testing: yes/no’ was modeled by logistic regression with age, HR+/HER2− subtype, family history and treatment line as independent variables. A *p*-value below 0.05 is viewed as statistically significant.

## 3. Results

### 3.1. Demographics

In total, 229 patients with a HER2− mBC were included in the study. The mean age of the study population was 64 ± 13 years. A total of 21.0% of patients had a positive family history for breast and/or ovarian cancer. The majority of patients (76.4%) had a HR+, HER2− mBC. Patients were on average on the second treatment line for the metastatic disease at the time of study inclusion (see Table 1).

### 3.2. Genetic Testing Rates

A total of 47.6% (109 of 229) of HER2− mBC patients had been advised to undergo genetic testing by the MTB. Of these informed patients, 89.0% (97 of 109) underwent genetic testing.

In total, 11.0% (12 of 109) of informed patients did not undergo genetic testing, 2 of these patients refused genetic testing after counseling, 1 patient died prior to genetic testing and in 9 patients the reason for not having undergone genetic testing was not explicit.

**Figure 1:**
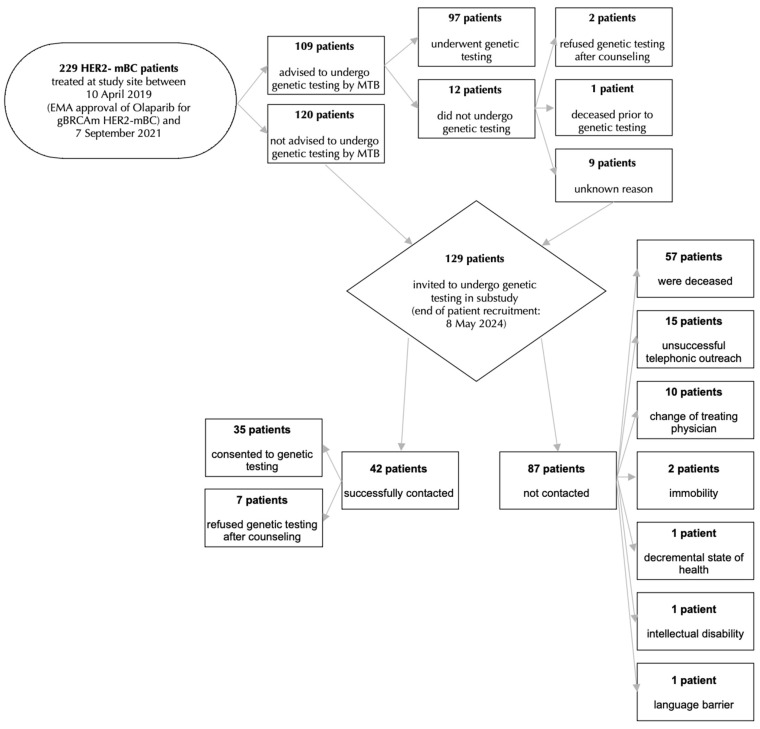
Study population overview. HER2−: Human Epidermal Growth Factor Receptor 2 negative, mBC: metastatic breast cancer, EMA: European Medicines Agency, gBRCAm: germline breast cancer gene mutant, MTB: multidisciplinary tumor board.

Overall, 52.4% (120 of 229) of HER2− mBC patients had not been recommended to undergo genetic testing by the MTB (see Figure 1).

### 3.3. Genetic Testing Rates in the Sub-Study

These 120 patients who had not been recommended to undergo genetic testing by the MTB as well as patients who had been advised to undergo genetic testing but had not for unknown reason (n = 9) were contacted in the course of a subsequent study and invited to undergo genetic testing.

In total, 42 of these 129 patients could successfully be contacted. Of these, 83.3% (35 of 42) of patients consented to genetic testing. However, 16.7% (7 of 42) of informed patients refused to undergo genetic testing.

A total of 87 patients in the subsequent study could not be informed and invited to genetic testing due to the following reasons: patient was deceased (n = 57), unsuccessful telephonic outreach (n = 15), change in treating physician (n = 10), immobility (n = 2), decremental state of health (n = 1), intellectual disability (n = 1) and language barrier (n = 1) (see Figure 1).

### 3.4. Predictors of Genetic Testing

In multivariate analysis the variables younger age (*p*-value: 0.0007), HR+/HER2− subtype (*p*-value < 0.0001) and positive family history for breast and ovarian cancer (*p*-value: 0.0001) were significantly associated with the performance of genetic counseling. Treatment line showed no association with the performance of genetic counseling (*p*-value 2nd line: 0.2292, *p*-value 3rd line: 0.68) (see Table 1).

### 3.5. Genetic Test Results

Genetic test results are shown for the retrospective cohort only. Information on genetic test results of the prospective subsequent study is still pending at the time of manuscript preparation and will be presented elsewhere.

The majority of patients had a WT or Variant of Uncertain Significance (VUS) result (84.2%). The most frequent pathogenic mutation was in the BRCA2 (8.4%), followed by BRCA1 (4.2%), partner and localizer of BRCA2 (PALB2) (1.1%) and Checkpoint kinase 2 (CHEK2) gene (1.1%). In 2 of 97 tested patients the genetic test result was unknown. An overview of genetic test results is displayed in Table 2.

### 3.6. PARP Inhibitor Treatment

Data on the uptake of PARPi treatment is shown for the retrospective cohort only. Information on the use of PARPis in the prospective subsequent study is still being collected at the time of manuscript preparation and will be published elsewhere.

In total, 11.6% (11 of 95) of tested patients had a gBRCA1/2mut and were therefore eligible for PARPi treatment, 4 patients had a gBRCA1mut and 7 patients a gBRCA2mut. 1 patient had a somatic (s) BRCA2mut (see Table 2).

Overall, 75% (9 of 12) of patients with a gBRCA or sBRCAmut had received treatment with a PARPi. The three patients with a gBRCAmut who had not received a PARPi were on an early treatment line for metastatic disease (1 patient with TN disease on 1st treatment line, 1 patient with HR+ disease on 1st treatment line and 1 patient with HR+ disease on 2nd treatment line).

2 HER2− mBC patients without a gBRCA or sBRCAmut were treated with a PARPi on the basis of compassionate use as well as in the course of a clinical study (see Table 3).

## 4. Discussion

Referral rates for genetic testing of BC patients who potentially qualify for PARPi treatment or could profit from platinum-based chemotherapy remain suboptimal despite guideline recommendations [11,12,13,24,25,26,27,29,30,31].

In the present retrospective analysis of a single academic center in Austria only 47.6% (109 of 229) of HER2− mBC patients had been recommended to undergo genetic testing by the MTB. Notably, informed patients showed a high acceptance rate to undergo genetic testing, with 93.6% (132 of 141) of patients willing to undergo genetic testing, compared to 6.4% (9 of 141) who refused genetic testing. Comparably low genetic testing rates have been reported by other real-world studies [25,32]. As such an analysis of the Flatiron database revealed that only 16.7% of over 12,000 patients with mBC (86% HR+/HER2− and 14% TNBC) had received genetic testing [32].

Particularly in recent years a significant decline in genetic testing rates has been reported which may be explained by the introduction of new targeted endocrine treatment options such as CDK4/6 inhibitors in HR+/HER2− mBC [28]. A retrospective analysis of BRCA1/2m HER2− mBC patients from the USA, European Union and Israel showed that endocrine therapies were the most prevalent in patients with HR+/HER2− mBC across all treatment lines (62%, 63% and 47% in 1st, 2nd and 3rd treatment line) [33]. In TNBC patients platinum-based chemotherapy was the most common therapy in first- and third-line patients, while non-platinum-based chemotherapy was the most frequent therapy in the second treatment line. The use of PARPis only increased in later treatment lines in both BC subtypes (HR+ subtype: 1st, 2nd and 3rd treatment line: 5%, 11% and 12%; TN subtype: 1st, 2nd and 3rd treatment line: 18%, 44% and 36%) [33].

In comparison, in the present study only 3.9% (9 of 229) of all HER2− mBC patients received PARPis and treatment line showed no association with the performance of genetic counseling in order to identify potential candidates for PARPis in multivariate analysis (*p*-value second line: 0.2292, *p*-value third line: 0.68).

The EMA label approves Olaparib as monotherapy for gBRCAm HER2− locally advanced or mBC patients who have previously received chemotherapy in the neoadjuvant, adjuvant or metastatic setting and in case of HR+ mBC were treated prior with endocrine therapy or were considered inappropriate for endocrine therapy. Yet, it is important to note that, the optimal sequencing of PARPis and endocrine-targeted treatments has not been established particularly in view of emerging evidence of better outcomes when PARPis are used earlier in the treatment cascade. As such, an exploratory subgroup analysis of the OlympiAD trial showed a greater OS benefit with the PARPi compared to TPC in the first-line setting, emphasizing the importance of early treatment initiation [4,34]. Likewise, in the phase IIIb LUCY trial median OS was longer in gBRCAm HER2− mBC patients who received first-line Olaparib than in second- or third-line settings [35]. Similar results of improved outcomes in cases of earlier implementation in the therapeutic algorithm were shown for other PARPis as well [36,37].

The indication for PARPis and therefore the need for genetic testing will become increasingly relevant as combinational treatments with other drug classes such as chemotherapy (NCT02163694), programmed cell death 1 (PD-1) or programmed cell death ligand 1 (PD-L1) immune checkpoint inhibitors (NCT02849496) as well as AKT inhibitors are being studied [20,36,37,38,39,40,41,42,43].

Moreover, pathogenic variants in BC susceptibility genes beyond BRCA1/2 are increasingly being considered in clinical trials with targeted therapies emphasizing the need for multigene panels [44,45,46,47]. As such PALB2 mutations have been identified as predictive markers for PARPi treatment [44]. Notably, identification of targetable variants and therefore treatment options were two times more likely following the extension of genetic testing to multigene panels [48,49,50]. Multigene panels for BC may include (but are not limited to) ATM, BARD1, BRCA1, BRCA2, CDH1, CHEK2, NF1, PALB2, PTEN, RAD51C, RAD51D, STK11 and TP53 [12,51].

In the present study—similar to other real-world data—11.6% (11 of 95) of tested patients had a gBRCAmut and were eligible for targeted treatment [7,8]. Notably, one patient had a sBRCA2mut and subsequently received PARPi treatment in the course of a compassionate use program.

There is accumulating evidence to support testing for sBRCA1/2muts as approximately 3% of BCs harbor exclusively sBRCA1/2mut and might benefit from targeted therapy (NCT03344965) [44,52,53,54,55]. Furthermore, tumor testing allows screening for additional alterations and treatment targets [56,57,58].

In the present study, younger age (*p*-value: 0.0007), HR+/HER2− subtype (*p*-value < 0.0001) and positive family history (*p*-value: 0.0001) were significantly associated with the performance of genetic counseling in multivariate analysis. While these parameters have been related with BRCAm BC—it is worth noting that in the PRAEGNANT registry, a German real-world registry for mBC patients, approximately 66% of patients with a BRCA1/2mut did not have a family history of BC [9]. In another study including breast and ovarian cancer patients 39% of BRCAm patients had no family history [59].

Notably, patients with an unknown family history are more likely to undergo BRCAmut testing if they have TNBC compared to HR+/HER2− disease [60]. The effect of BC subtype on genetic testing rates was shown to be especially pronounced in non-academic settings [28,61].

While there is a relatively high prevalence of BRCAmuts in TN subtype, a multinational epidemiologic study among BRCA1mut carriers showed that 22% of tumors were estrogen receptor positive (ER+) and 21% were progesterone receptor positive (PR+), while among BRCA2mut carriers, 77% were ER+ and 64% were PR+ [62]. Therefore, genetic testing must be considered in HR+ disease if other risk factors for hereditary disease are present or PARPis and platinum salts are treatment options.

It is worth noting that the benefit with Olaparib in OlympiAD was independent of HR status [34]. Likewise, the ABRAZO study showed similar response rates to Talazoparib in BRCA1/2mut carriers with mBC independent of subtype (ER+: 29% versus TN: 26%) [63].

In the present study—besides the lack of recommendation by the MTB (52.4% (120/229) of the total patient population) and patients’ refusal to undergo genetic testing (6.4% (9/141) of informed patients)— genetic testing was not performed due to immobility of the patient (1.6% (2/129) of untested patients were not informed and invited to undergo genetic testing) and language barrier of the patient (0.8% (1/129) of untested patients were not informed and invited to undergo genetic testing).

This emphasizes the need to improve education on hereditary breast and ovarian cancer and introduce alternative genetic counseling strategies to increase access to genetic testing.

Notably, genetic counseling by a physician or geneticist is a prerequisite for genetic testing performance. The physician has been shown to have significant impact on whether patients consent to genetic testing [64]. Therefore, improved education of physicians, increasing their awareness of testing indications, clinical consequences and therapeutic options of hereditary breast and ovarian cancer and therefore their perception of the importance of genetic testing might subsequently increase its uptake.

Furthermore, besides mainstream testing approaches, where not only a geneticist but various members of the medical oncology team perform genetic counseling and testing [65], the introduction of digital tools including web-based tools, mobile applications, chatbots, videos and games in the counseling process has been shown to improve access to genetic testing [66,67,68,69,70].

The present study has some limitations. Firstly, due to the retrospective nature, no information on the incentive for genetic testing such as HER2− metastatic disease and/or family history is available. Secondly, the study was performed in a single academic institution. A multinational and institutional setting might lead to different results.

## 5. Conclusions

The present study—in line with other real-world data—demonstrated suboptimal referral rates of HER2− mBC patients in an academic setting, especially in older patients, those with a HR+ subtype and no family history of breast and ovarian cancer. While young age, TN subtype and positive family history are associated with BRCAm BC–genetic testing should not be limited to these patient populations if additional risk factors for hereditary disease or a potential indication for a PARPi and platinum-based chemotherapy are present. Measures to increase awareness for genetic testing indications as well as new genetic counseling strategies to increase access to genetic testing are needed. Particularly, as the present study demonstrated that patients who had been counseled showed a high willingness to undergo genetic testing which revealed targetable mutations in over 10% of tested patients.

## Figures and Tables

**Table 1:** Demographic data and predictive markers for genetic testing performance.

Patient Characteristics	Total Study Population	Recommendation for Genetic Testing		*p*-Value
	Number of Patients (%, n = 229)	Yes Number of Patients (%, n = 109)	No Number of Patients (%, n = 120)	
**Age**, mean ± SD	64 ± 13	59 ± 13.4	67.6 ± 11.8	0.0007
**Breast cancer subtype**				0.000
- HR+/HER2−	76.4	55.0	95.8	
- TN	23.6	45.0	4.2	
**Treatment line**				
- 1st treatment line	52.8	50.5	55	
- 2nd treatment line	17.5	16.5	18.3	0.2292
≥3rd treatment line	29.7	33.0	26.7	0.68
- mean ± SD	2.0 ± 1.5	1.0 ± 1.6	1.0 ± 1.3	
**Family history**				0.0001
- positive	21.0	33.0	10.0	
- negative	51.1	45.0	56.7	
- unknown	27.9	22.0	33.3	

SD: standard deviation, HR+: hormone receptor positive, HER2−: Human Epidermal Growth Factor Receptor 2 negative, TN: triple negative.

**Table 2:** Genetic test results (only including the 95 patients with a known genetic test result).

Genetic Test Result	Number of Patients (%, n = 95)
WT or VUS	84.2
BRCA2 1 patient with a somatic mutation (only tumor tissue was tested in this patient.)	8.4
BRCA1	4.2
PALB2	1.1
CHEK2	1.1

WT: wild-type, VUS: variant of uncertain significance, BRCA1/2: breast cancer gene 1/2, PALB2: partner and localizer of BRCA2, CHEK2: Checkpoint kinase 2.

**Table 3:** PARP inhibitor treatment.

Treatment Indication	Absolute Number of Patients
**Targetable mutations**	**9**
- gBRCA1/2mut	8
- sBRCA2mut	1
**No targetable mutations**	**2**
- Compassionate use	1
- Clinical study	1

gBRCAmut: germline breast cancer gene mutation, sBRCAmut: somatic breast cancer gene mutation.

## Data Availability

Research data includes confidential patient information and is therefore unavailable for distribution.

## References

[1] Pommier Y., O’Connor M.J., de Bono J. (2016). Laying a trap to kill cancer cells: PARP inhibitors and their mechanisms of action. Sci. Transl. Med..

[2] Robson M., Im S.A., Senkus E., Xu B., Domchek S.M., Masuda N., Delaloge S., Li W., Tung N., Armstrong A. (2017). Olaparib for Metastatic Breast Cancer in Patients with a Germline BRCA Mutation. N. Engl. J. Med..

[3] Litton J.K., Rugo H.S., Ettl J., Hurvitz S.A., Goncalves A., Lee K.H., Fehrenbacher L., Yerushalmi R., Mina L.A., Martin M. (2018). Talazoparib in Patients with Advanced Breast Cancer and a Germline BRCA Mutation. N. Engl. J. Med..

[4] Robson M.E., Im S.A., Senkus E., Xu B., Domchek S.M., Masuda N., Delaloge S., Tung N., Armstrong A., Dymond M. (2023). OlympiAD extended follow-up for overall survival and safety: Olaparib versus chemotherapy treatment of physician’s choice in patients with a germline BRCA mutation and HER2−negative metastatic breast cancer. Eur. J. Cancer.

[5] Litton J.K., Hurvitz S.A., Mina L.A., Rugo H.S., Lee K.H., Goncalves A., Diab S., Woodward N., Goodwin A., Yerushalmi R. (2020). Talazoparib versus chemotherapy in patients with germline BRCA1/2-mutated HER2−negative advanced breast cancer: Final overall survival results from the EMBRACA trial. Ann. Oncol..

[6] Ettl J., Quek R.G.W., Lee K.H., Rugo H.S., Hurvitz S., Goncalves A., Fehrenbacher L., Yerushalmi R., Mina L.A., Martin M. (2018). Quality of life with talazoparib versus physician’s choice of chemotherapy in patients with advanced breast cancer and germline BRCA1/2 mutation: Patient-reported outcomes from the EMBRACA phase III trial. Ann. Oncol..

[7] Armstrong N., Ryder S., Forbes C., Ross J., Quek R.G. (2019). A systematic review of the international prevalence of BRCA mutation in breast cancer. Clin. Epidemiol..

[8] O’Shaughnessy J., Brezden-Masley C., Cazzaniga M., Dalvi T., Walker G., Bennett J., Ohsumi S. (2020). Prevalence of germline BRCA mutations in HER2−negative metastatic breast cancer: Global results from the real-world, observational BREAKOUT study. Breast Cancer Res..

[9] Fasching P.A., Yadav S., Hu C., Wunderle M., Haberle L., Hart S.N., Rubner M., Polley E.C., Lee K.Y., Gnanaolivu R.D. (2021). Mutations in BRCA1/2 and Other Panel Genes in Patients with Metastatic Breast Cancer -Association with Patient and Disease Characteristics and Effect on Prognosis. J. Clin. Oncol..

[10] Fasching P.A., Hu C., Hart S.N., Polley E.C., Lee K.Y., Gnanolivu R.D., Lilyquist J., Hartkopf A.D., Taran F.A., Janni W. (2018). HartSNet Cancer predisposition genes in metastatic breast cancer—Association with metastatic pattern, prognosis, patient and tumor characteristics. Cancer Res..

[11] Bedrosian I., Somerfield M.R., Achatz M.I., Boughey J.C., Curigliano G., Friedman S., Kohlmann W.K., Kurian A.W., Laronga C., Lynce F. (2024). Germline Testing in Patients with Breast Cancer: ASCO-Society of Surgical Oncology Guideline. J. Clin. Oncol..

[12] NCCN Clinical Practice Guidelines in Oncology (NCCN Guidelines^®^) (2024). Genetic/Familial High-Risk Assessment: Breast, O., and Pancreatic V.3.2024.

[13] (2024). 2023, Early Breast Cancer Clinical Practice Guideline. https://data.esmo.org/guidelines/pdf/ESMO_2023_BreastCancer.html.

[14] Bhattacharyya A., Ear U.S., Koller B.H., Weichselbaum R.R., Bishop D.K. (2000). The breast cancer susceptibility gene BRCA1 is required for subnuclear assembly of Rad51 and survival following treatment with the DNA cross-linking agent cisplatin. J. Biol. Chem..

[15] Lord C.J., Ashworth A. (2016). BRCAness revisited. Nat. Rev. Cancer.

[16] Tutt A., Tovey H., Cheang M.C.U., Kernaghan S., Kilburn L., Gazinska P., Owen J., Abraham J., Barrett S., Barrett-Lee P. (2018). Carboplatin in BRCA1/2-mutated and triple-negative breast cancer BRCAness subgroups: The TNT Trial. Nat. Med..

[17] Tutt A., Ellis P., Kilburn L., Gilett C., Pinder S., Abraham J., Barrett S., Barrett-Lee P., Chan S., Cheang M. (2015). The TNT trial: A randomized phase III trial of carboplatin (C) compared with docetaxel (D) for patients with metastatic or recurrent locally advanced triple negative or BRCA1/2 breast cancer (CRUK/07/012). Cancer Res..

[18] Byrski T., Dent R., Blecharz P., Foszczynska-Kloda M., Gronwald J., Huzarski T., Cybulski C., Marczyk E., Chrzan R., Eisen A. (2012). Results of a phase II open-label, non-randomized trial of cisplatin chemotherapy in patients with BRCA1-positive metastatic breast cancer. Breast Cancer Res..

[19] Isakoff S.J., Mayer E.L., He L., Traina T.A., Carey L.A., Krag K.J., Rugo H.S., Liu M.C., Stearns V., Come S.E. (2015). TBCRC009: A Multicenter Phase II Clinical Trial of Platinum Monotherapy with Biomarker Assessment in Metastatic Triple-Negative Breast Cancer. J. Clin. Oncol..

[20] Han H.S., Dieras V., Robson M., Palacova M., Marcom P.K., Jager A., Bondarenko I., Citrin D., Campone M., Telli M.L. (2018). Veliparib with temozolomide or carboplatin/paclitaxel versus placebo with carboplatin/paclitaxel in patients with BRCA1/2 locally recurrent/metastatic breast cancer: Randomized phase II study. Ann. Oncol..

[21] Tung N.M., Garber J.E. (2018). BRCA1/2 testing: Therapeutic implications for breast cancer management. Br. J. Cancer.

[22] Goodwin P.J., Phillips K.A., West D.W., Ennis M., Hopper J.L., John E.M., O’Malley F.P., Milne R.L., Andrulis I.L., Friedlander M.L. (2012). Breast cancer prognosis in BRCA1 and BRCA2 mutation carriers: An International Prospective Breast Cancer Family Registry population-based cohort study. J. Clin. Oncol..

[23] Liu M., Xie F., Liu M., Zhang Y., Wang S. (2021). Association between BRCA mutational status and survival in patients with breast cancer: A systematic review and meta-analysis. Breast Cancer Res. Treat..

[24] Tung N., Battelli C., Allen B., Kaldate R., Bhatnagar S., Bowles K., Timms K., Garber J.E., Herold C., Ellisen L. (2015). Frequency of mutations in individuals with breast cancer referred for BRCA1 and BRCA2 testing using next-generation sequencing with a 25-gene panel. Cancer.

[25] Childers C.P., Childers K.K., Maggard-Gibbons M., Macinko J. (2017). National Estimates of Genetic Testing in Women with a History of Breast or Ovarian Cancer. J. Clin. Oncol..

[26] Lux M.P., Lewis K., Rider A., Niyazov A. (2022). Treatment Patterns, Safety, and Patient Reported Outcomes among Adult Women with Human Epidermal Growth Factor Receptor 2-Negative Advanced Breast Cancer with or without, or with Unknown, BRCA1/2 Mutation(s): Results of a Real-World Study from the United States, United Kingdom, and four EU Countries. Breast Care.

[27] Dalvi T., McLaurin K., Briceno J., Nordstrom B., Bennett J., Hettle R., Murphy B., Collins J., McCutcheon S. (2018). A Real World Evidence Study of BRCA mutations and survival in HER2−negative breast cancer. Cancer Res..

[28] Lux M.P., Lewis K., Rider A., Niyazov A. (2022). Real-world multi-country study of BRCA1/2 mutation testing among adult women with HER2−negative advanced breast cancer. Future Oncol..

[29] Brugioni E., Cathcart-Rake E., Metsker J., Gustafson E., Douglass L., Pluard T.J. (2023). Germline BRCA-Mutated HER2−Negative Advanced Breast Cancer: Overcoming Challenges in Genetic Testing and Clinical Considerations When Using Talazoparib. Clin. Breast Cancer.

[30] Kemp Z., Turnbull A., Yost S., Seal S., Mahamdallie S., Poyastro-Pearson E., Warren-Perry M., Eccleston A., Tan M.M., Teo S.H. (2019). Evaluation of Cancer-Based Criteria for Use in Mainstream BRCA1 and BRCA2 Genetic Testing in Patients with Breast Cancer. JAMA Netw. Open.

[31] Mahtani R., Niyazov A., Lewis K., Rider A., Massey L., Arondekar B., Lux M.P. (2023). Real-World Study of Regional Differences in Patient Demographics, Clinical Characteristics, and BRCA1/2 Mutation Testing in Patients with Human Epidermal Growth Factor Receptor 2-Negative Advanced Breast Cancer in the United States, Europe, and Israel. Adv. Ther..

[32] Quek R.G.W., Mardekian J. (2019). Clinical Outcomes, Treatment Patterns, and Health Resource Utilization Among Metastatic Breast Cancer Patients with Germline BRCA1/2 Mutation: A Real-World Retrospective Study. Adv. Ther..

[33] Mahtani R., Niyazov A., Arondekar B., Lewis K., Rider A., Massey L., Lux M.P. (2022). Real-world study of patients with germline BRCA1/2-mutated human epidermal growth factor receptor 2‒Negative advanced breast cancer: Patient demographics, treatment patterns, adverse events, and physician-reported satisfaction in the United States, Europe, and Israel. Breast.

[34] Senkus E., Delaloge S., Domchek S.M., Conte P., Im S.A., Xu B., Armstrong A., Masuda N., Fielding A., Robson M. (2023). Olaparib efficacy in patients with germline BRCA-mutated, HER2−negative metastatic breast cancer: Subgroup analyses from the phase III OlympiAD trial. Int. J. Cancer.

[35] Balmana J., Fasching P.A., Couch F.J., Delaloge S., Labidi-Galy I., O’Shaughnessy J., Park Y.H., Eisen A.F., You B., Bourgeois H. (2024). Clinical effectiveness and safety of olaparib in BRCA-mutated, HER2−negative metastatic breast cancer in a real-world setting: Final analysis of LUCY. Breast Cancer Res. Treat..

[36] Domchek S.M., Postel-Vinay S., Im S.A., Park Y.H., Delord J.P., Italiano A., Alexandre J., You B., Bastian S., Krebs M.G. (2020). Olaparib and durvalumab in patients with germline BRCA-mutated metastatic breast cancer (MEDIOLA): An open-label, multicentre, phase 1/2, basket study. Lancet Oncol..

[37] Vinayak S., Tolaney S.M., Schwartzberg L., Mita M., McCann G., Tan A.R., Wahner-Hendrickson A.E., Forero A., Anders C., Wulf G.M. (2019). Open-label Clinical Trial of Niraparib Combined with Pembrolizumab for Treatment of Advanced or Metastatic Triple-Negative Breast Cancer. JAMA Oncol..

[38] Somlo G., Frankel P.H., Arun B.K., Ma C.X., Garcia A.A., Cigler T., Cream L.V., Harvey H.A., Sparano J.A., Nanda R. (2017). Efficacy of the PARP Inhibitor Veliparib with Carboplatin or as a Single Agent in Patients with Germline BRCA1- or BRCA2-Associated Metastatic Breast Cancer: California Cancer Consortium Trial NCT01149083. Clin. Cancer Res..

[39] Balmana J., Tung N.M., Isakoff S.J., Grana B., Ryan P.D., Saura C., Lowe E.S., Frewer P., Winer E., Baselga J. (2014). Phase I trial of olaparib in combination with cisplatin for the treatment of patients with advanced breast, ovarian and other solid tumors. Ann. Oncol..

[40] Del Conte G., Sessa C., von Moos R., Vigano L., Digena T., Locatelli A., Gallerani E., Fasolo A., Tessari A., Cathomas R. (2014). Phase I study of olaparib in combination with liposomal doxorubicin in patients with advanced solid tumours. Br. J. Cancer.

[41] Samol J., Ranson M., Scott E., Macpherson E., Carmichael J., Thomas A., Cassidy J. (2012). Safety and tolerability of the poly(ADP-ribose) polymerase (PARP) inhibitor, olaparib (AZD2281) in combination with topotecan for the treatment of patients with advanced solid tumors: A phase I study. Investig. New Drugs.

[42] Rugo H.S., Dent R., Kim S.-B., Dalenc F., Ruiz E.Y., Im S.-H. (2024). Pembrolizumab + olaparib vs pembrolizumab + chemotherapy after induction with pembrolizumab + chemotherapy for locally recurrent inoperable or metastatic TNBC: Randomized open-label Phase 2 KEYLYNK-009 study. Cancer Res..

[43] Domchek S., Postel-Vinay S., Bang Y.-J., Park Y., Alexandre J., Delord J.-P., Italiano A., You B., Bastian S., Krebs M. (2018). An open-label, multitumor, phase II basket study of olaparib and durvalumab (MEDIOLA): Results in germline BRCA-mutated (gBRCAm) HER2−negative metastatic breast cancer (MBC). Cancer Res..

[44] Tung N.M., Robson M.E., Ventz S., Santa-Maria C.A., Nanda R., Marcom P.K., Shah P.D., Ballinger T.J., Yang E.S., Vinayak S. (2020). TBCRC 048: Phase II Study of Olaparib for Metastatic Breast Cancer and Mutations in Homologous Recombination-Related Genes. J. Clin. Oncol..

[45] Gruber J.J., Afghahi A., Timms K., DeWees A., Gross W., Aushev V.N., Wu H.T., Balcioglu M., Sethi H., Scott D. (2022). A phase II study of talazoparib monotherapy in patients with wild-type BRCA1 and BRCA2 with a mutation in other homologous recombination genes. Nat. Cancer.

[46] Eikesdal H.P., Yndestad S., Elzawahry A., Llop-Guevara A., Gilje B., Blix E.S., Espelid H., Lundgren S., Geisler J., Vagstad G. (2021). Olaparib monotherapy as primary treatment in unselected triple negative breast cancer. Ann. Oncol..

[47] Burstein H.J., Somerfield M.R., Barton D.L., Dorris A., Fallowfield L.J., Jain D., Johnston S.R.D., Korde L.A., Litton J.K., Macrae E.R. (2021). Endocrine Treatment and Targeted Therapy for Hormone Receptor-Positive, Human Epidermal Growth Factor Receptor 2-Negative Metastatic Breast Cancer: ASCO Guideline Update. J. Clin. Oncol..

[48] Fanale D., Incorvaia L., Filorizzo C., Bono M., Fiorino A., Calo V., Brando C., Corsini L.R., Barraco N., Badalamenti G. (2020). Detection of Germline Mutations in a Cohort of 139 Patients with Bilateral Breast Cancer by Multi-Gene Panel Testing: Impact of Pathogenic Variants in Other Genes beyond BRCA1/2. Cancers.

[49] Bono M., Fanale D., Incorvaia L., Cancelliere D., Fiorino A., Calo V., Dimino A., Filorizzo C., Corsini L.R., Brando C. (2021). Impact of deleterious variants in other genes beyond BRCA1/2 detected in breast/ovarian and pancreatic cancer patients by NGS-based multi-gene panel testing: Looking over the hedge. ESMO Open.

[50] Kurian A.W., Ward K.C., Hamilton A.S., Deapen D.M., Abrahamse P., Bondarenko I., Li Y., Hawley S.T., Morrow M., Jagsi R. (2018). Uptake, Results, and Outcomes of Germline Multiple-Gene Sequencing After Diagnosis of Breast Cancer. JAMA Oncol..

[51] Kurian A.W., Ward K.C., Howlader N., Deapen D., Hamilton A.S., Mariotto A., Miller D., Penberthy L.S., Katz S.J. (2019). Genetic Testing and Results in a Population-Based Cohort of Breast Cancer Patients and Ovarian Cancer Patients. J. Clin. Oncol..

[52] Walsh E.M., Mangini N., Fetting J., Armstrong D., Chan I.S., Connolly R.M., Fiallos K., Lehman J., Nunes R., Petry D. (2022). Olaparib Use in Patients with Metastatic Breast Cancer Harboring Somatic BRCA1/2 Mutations or Mutations in Non-BRCA1/2, DNA Damage Repair Genes. Clin. Breast Cancer.

[53] Batalini F., Madison R., Pavlick D.C., Sokol E., Snow T., Sondhi A., Frampton G.M., Jenkins C., Garber J.E., Wulf G.M. (2021). Analysis of real-world (RW) data for metastatic breast cancer (mBC) patients (pts) with somatic BRCA1/2 (sBRCA) or other homologous recombination (HR)-pathway gene mutations (muts) treated with PARP inhibitors (PARPi). J. Clin. Oncol..

[54] Blum J.L., Laird A.D., Litton J.K., Rugo H.S., Ettl J., Hurvitz S.A., Martin M., Roché H.H., Lee K.-H., Goodwin A. (2022). Determinants of Response to Talazoparib in Patients with HER2-Negative, Germline BRCA1/2-Mutated Breast Cancer. Clin. Cancer Res..

[55] Hodgson D., Lai Z., Dearden S., Barrett J., Harrington E., Timms K., Lanchbury J., Wu W., Allen A., Senkus E. (2021). Analysis of mutation status and homologous recombination deficiency in tumors of patients with germline BRCA1 or BRCA2 mutations and metastatic breast cancer: OlympiAD. Ann. Oncol..

[56] Polak P., Kim J., Braunstein L.Z., Karlic R., Haradhavala N.J., Tiao G., Rosebrock D., Livitz D., Kubler K., Mouw K.W. (2017). A mutational signature reveals alterations underlying deficient homologous recombination repair in breast cancer. Nat. Genet..

[57] Lai Z., Brosnan M., Sokol E.S., Xie M., Dry J.R., Harrington E.A., Barrett J.C., Hodgson D. (2022). Landscape of homologous recombination deficiencies in solid tumours: Analyses of two independent genomic datasets. BMC Cancer.

[58] Zehir A., Benayed R., Shah R.H., Syed A., Middha S., Kim H.R., Srinivasan P., Gao J., Chakravarty D., Devlin S.M. (2017). Mutational landscape of metastatic cancer revealed from prospective clinical sequencing of 10,000 patients. Nat. Med..

[59] Brozek I., Ratajska M., Piatkowska M., Kluska A., Balabas A., Dabrowska M., Nowakowska D., Niwinska A., Rachtan J., Steffen J. (2012). Limited significance of family history for presence of BRCA1 gene mutation in Polish breast and ovarian cancer cases. Fam. Cancer.

[60] Lux M.P., Decker T., Runkel E.D., Niyazov A., Quek R.G.W., Marschner N., Harbeck N. (2022). Awareness and Availability of Routine Germline BRCA1/2 Mutation Testing in Patients with Advanced Breast Cancer in Germany. Breast Care.

[61] Mahtani R.L., Niyazov A., Lewis K., Briceno J., Nordstrom B., Bennett J., Hettle R. (2020). Germline BRCA1/2 (gBRCA1/2) testing patterns among oncologists (ONC) treating HER2− advanced breast cancer (ABC): Results from a multi-country real-world study. Ann. Oncol..

[62] Mavaddat N., Barrowdale D., Andrulis I.L., Domchek S.M., Eccles D., Nevanlinna H., Ramus S.J., Spurdle A., Robson M., Sherman M. (2012). Pathology of breast and ovarian cancers among BRCA1 and BRCA2 mutation carriers: Results from the Consortium of Investigators of Modifiers of BRCA1/2 (CIMBA). Cancer Epidemiol. Biomark. Prev..

[63] Turner N.C., Telli M.L., Rugo H.S., Mailliez A., Ettl J., Grischke E.-M., Mina L.A., Gelpi J.B., Fasching P.A., Hurvitz S.A. (2017). Final results of a phase 2 study of talazoparib (TALA) following platinum or multiple cytotoxic regimens in advanced breast cancer patients (pts) with germline BRCA1/2 mutations (ABRAZO). J. Clin. Oncol..

[64] Katz S.J., Bondarenko I., Ward K.C., Hamilton A.S., Morrow M., Kurian A.W., Hofer T.P. (2018). Association of Attending Surgeon with Variation in the Receipt of Genetic Testing After Diagnosis of Breast Cancer. JAMA Surg..

[65] Bokkers K., Frederix G., Velthuizen M., van der Aa M., Gerestein C., van Dorst E., Lange J., Louwers J., Koole W., Zweemer R. (2022). Mainstream germline genetic testing for patients with epithelial ovarian cancer leads to higher testing rates and a reduction in genetics-related healthcare costs from a healthcare payer perspective. Gynecol. Oncol..

[66] George A., Riddell D., Seal S., Talukdar S., Mahamdallie S., Ruark E., Cloke V., Slade I., Kemp Z., Gore M. (2016). Implementing rapid, robust, cost-effective, patient-centred, routine genetic testing in ovarian cancer patients. Sci. Rep..

[67] Schwartz M.D., Valdimarsdottir H.B., Peshkin B.N., Mandelblatt J., Nusbaum R., Huang A.T., Chang Y., Graves K., Isaacs C., Wood M. (2014). Randomized noninferiority trial of telephone versus in-person genetic counseling for hereditary breast and ovarian cancer. J. Clin. Oncol..

[68] Shickh S., Rafferty S.A., Clausen M., Kodida R., Mighton C., Panchal S., Lorentz J., Ward T., Watkins N., Elser C. (2021). The role of digital tools in the delivery of genomic medicine: Enhancing patient-centered care. Genet. Med..

[69] Bombard Y., Hayeems R.Z. (2020). How digital tools can advance quality and equity in genomic medicine. Nat. Rev. Genet..

[70] Lee W., Shickh S., Assamad D., Luca S., Clausen M., Somerville C., Tafler A., Shaw A., Hayeems R., Bombard Y. (2023). Patient-facing digital tools for delivering genetic services: A systematic review. J. Med. Genet..

